# Smallpox Models as Policy Tools^1^

**DOI:** 10.3201/eid1011.040455

**Published:** 2004-11

**Authors:** F. Ellis McKenzie

**Affiliations:** * National Institutes of Health, Bethesda, Maryland, USA

**Keywords:** models, policy, biodefense, perspective

## Abstract

Mathematical models can help prepare for and respond to bioterrorism attacks, provided that their strengths and weaknesses are clearly understood. A series of initiatives within the Department of Health and Human Services brought modelers together with biologists and epidemiologists who specialize in smallpox and experts in bioterrorism response and health policy and has led to the parallel development of models with different technical approaches but standardized scenarios, parameter ranges, and outcome measures. Cross-disciplinary interactions throughout the process supported the development of models focused on systematically comparing alternate intervention strategies, determining the most important issues in decision-making, and identifying gaps in current knowledge.

## Abstract 2

Interactions between policymakers and mathematical modelers can improve biodefense strategies.

The man who demonstrated that malaria is transmitted by mosquitoes, Sir Ronald Ross, developed the first mathematical model of malaria transmission in 1911. In presenting his model, Ross made the crucial point that "the mathematical method of treatment is really nothing but the application of careful reasoning to the problems at issue" (*1*). In short, mathematical modeling is no more and no less than a tool to support clear thinking.

In the United States, mathematical models are familiar, everyday tools in engineering, business, and military applications and in most sciences. They represent hypotheses about underlying mechanisms that generate observed phenomena or the options for action and potential consequences. However, those models are rare in the biomedical-research and public health communities.

The events of September 11, 2001, emphasized that the United States should use every tool available to help prepare for, and respond to, bioterrorism. With that understanding in mind, a series of National Institutes of Health (NIH) consultations was organized to address the potential of mathematical models to help with bioterrorism preparedness and response.

The first of those, in December 2001, brought together a small group of modelers and a small group of health-policy experts. The basic idea of this meeting was to see if a productive dialogue would emerge, and one did, despite the language and culture barriers. This dialogue led to a much better understanding of what modeling could and could not do to help.

The overall conclusion of the meeting was that models can be of great value, provided that their strengths and weaknesses are clearly understood. Modelers and nonmodelers should develop realistic expectations. For instance, models will not provide accurate numerical predictions of outcomes in this context; models can be used to forecast only in fairly gross terms. The key is to look not for absolute numbers but for differences in outcomes between different strategies and between different models. The consensus from that first consultation was that models can provide a means to systematically compare alternative intervention strategies, determine the most important issues in decision-making, and identify critical gaps in current knowledge.

Those three points are not as simple and straightforward as they may seem. For instance, if modeling is going to help identify and focus on the decisions likely to have the largest effects on outcomes, the models must address actual decisions to be made in actual bioterrorism events. That first consultation highlighted the need for active engagement and creative tension between modelers and policy experts. Modelers may focus on areas that interest them but seem tangential to decision-makers. On the other hand, if only policy experts are engaged, they may concentrate on information that fits their opinions and interests. The modeling most likely to help with bioterrorism preparedness and response will emerge from scientific, operational, and policy professionals who listen to and engage each other, with real respect and candor, on a continuing basis.

A corollary conclusion from that first consultation was that modeling can provide a comprehensive, explicit examination of the assumptions and logic that enter into a decision, in a way that purely verbal reasoning and debate cannot. In that sense, even if the results of a model were discarded, the modeling process alone, properly conducted, would more than return the investment.

Another way of looking at this same set of issues is the observation that many people, modelers and nonmodelers alike, seem to believe that one "right" model exists. In this context, at least, that is not likely to be the case. However, a great deal can be learned from examining circumstances in which several models disagree, whether or not they agree on some overall, qualitative result.

For example, the Figure shows output from two hypothetical models. The horizontal axis gives the fraction of a population covered by some intervention, e.g., a vaccine, and the vertical axis shows the resulting percentage reduction in death rate. At 0% coverage, the number of deaths does not change. Approaching 100% coverage, deaths are reduced nearly 100%. Both models agree that fewer deaths occur when more people are covered, but obvious differences also exist between the model results. According to model A, slightly less than 30% coverage would reduce deaths by half; according to model B, almost 70% coverage is needed to reduce deaths by the same amount.

**Figure:**
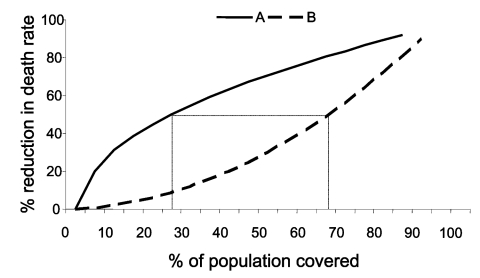
Output from two hypothetical models.

Because these sorts of models embody hypotheses about underlying mechanisms, the differences may have to do with varying ideas about how a particular vaccine works in particular subpopulations, at particular sites, with different methods of introduction, or whether the vaccine acts synergistically with some other intervention. Many possibilities and uncertainties exist, but each of those ideas is in the models, explicitly. One should be able to clearly see what the different assumptions are, why the modelers put them there, and what data support them.

The results to rely on, of course, are those on which a number of different models agree in general terms, not precise, detailed predictions. But if models disagree, if one assumes that they were created by competent, honest modelers, the information that must be used to make the decision comes under more scrutiny. What are the assumptions? What are the most critical gaps in the data? Why is there disagreement? Reasonable differences in assumptions that give rise to a critical difference in outcomes point to high priorities for research.

One of the recommendations from the first consultation was that a "proof of principle" project be undertaken for a specific set of issues. That recommendation was the basis of the second consultation, in April 2002, which focused specifically on smallpox modeling. The basic idea was to get a group of smallpox modelers in the same room to talk with smallpox biology and epidemiology experts and with bioterrorism-response and health-policy experts. A number of questions arose, but three stand out as examples of ways in which modeling can help to clarify assumptions.

The first of those questions was, "When does a person infected with smallpox become infectious? Is he or she still mobile, or already severely ill?" Joel Breman and D.A. Henderson had just published a review article (*2*), which stated, "Patients are most infectious from the onset of the enanthema through the first 7 to 10 days of rash." That is, the period of peak infectivity starts 1 day before the rash appears. Modelers interpreted that statement, and others in the literature, in a variety of ways, with slight differences in some cases and major differences in others. Differences in interpretation contributed to some of the more striking differences in model outcomes, which flagged the question as a critical one.

What is known about the infectivity of smallpox should be understood and represented in the most precise, accurate, and useful way possible. Modeling forces specific questions that help that process. If infected persons are less infectious before the onset of enanthema, how long before and how much less? Do onset and intensity of infectivity vary? By how much? How can infectivity or exposure be interpreted in terms of duration and distance of contact? The process of modeling forces an examination of the sensitivity of the results to specific answers.

The second question was, "What, in concrete, operational terms, is meant by ‘ring' vaccination?" Some participants based their understanding primarily on their own experience, some on reports in the literature, and some on the Centers for Disease Control and Prevention (CDC) interim response policy of that time. Discussions of how the models had translated this first-line response strategy, now usually known as "surveillance and containment," highlighted several discrepancies and ambiguities. Similar questions arose about the meanings of "isolation" and "quarantine."

Constructing mathematical models helps make premises explicit and quantifiable, to explain what is intended by concepts such as infectivity or ring vaccination. Models are tools, but mathematical models, more than purely verbal models, facilitate comprehensiveness and precision in describing assumptions and their implications.

The third question was, "How can models best represent the process by which an infectious agent may be transmitted?" The classic method of modeling considers a population to be divided into distinct subpopulations: susceptible, infected, infectious, and removed (i.e., dead or recovered and immune). Members of the population mix freely with each other, and disease spreads through contact between persons in the susceptible and infectious compartments. These models typically take the form of differential equations.

Computers have made possible a different approach to infectious disease modeling that allows modeling of interactions between distinct persons, some of whom may have many contacts during a given time period, while others have only a few. Again, differences in outcomes can arise from differences in assumptions, in this case, assumptions about social structures and mixing processes. And again, hazards of oversimplicity and overcomplexity can be remarkably subtle. For critical applications, the sensible move is to examine the sensitivity of results to specific methods by comparing different intervention strategies not only within each modeling framework but across different modeling frameworks.

Following the second consultation, the Secretary's Advisory Council on Public Health Preparedness, in the Department of Health and Human Services, formed a working group on smallpox modeling with a similar mix of people. The group was charged with developing models of smallpox spread and the potential effects of several types of interventions, in several attack scenarios, to help analyze a range of options. Specific tasks were for the working group as a whole to standardize scenarios, parameter ranges, and outcome measures. Then, modelers with different approaches (deterministic differential-equation, stochastic simulation, and individual-based simulation) were charged with developing draft models to be reviewed by the entire group, revised by the modelers, reviewed again, revised again, and so forth.

Each group of modelers is now nearly ready to submit a paper describing its model and results for peer review and publication; these articles will be accompanied by a detailed description of the common scenarios, assumptions, and parameter ranges. The aim here is to briefly outline the process the entire group went through and some of the factors considered in the models. This process has been unique in U.S. public health experience.

Achieving consensus on the scenarios and outcome measures was not difficult, at least not in comparison to the challenge of reviewing data and expert judgments and agreeing on parameter ranges and other assumptions. For all biology-epidemiology parameters, for example, ranges were expected to reflect what is known about the natural spread of smallpox, but existing information comes from efforts to treat, impede, and ultimately eradicate smallpox; the data were not collected to guide modelers, researchers, and policymakers. Thus, like most models, the models developed by the working group encompass a mixture of facts and hypotheses about mechanisms driving the dynamics at almost every level. As a result, as with most models, sensitivity analyses, which show the extent to which changes in parameters change results, make the caveats explicit and precise and also show which unknowns are most important to outcomes and most critical for research.

For example, the working group had to decide on probability distributions for the timing and intensity of key events such as incubation period, onset of fever and rash, onset and degrees of infectivity, and the like. While these discussions were often framed in biologic and clinical terms, in operational terms, the objective was to assess probabilities of case recognition, and in epidemiologic terms, the objective was to determine probabilities of transmission with various sorts of contact, to define "contact," and when possible, to calibrate everything to agreed-upon data in an agreed-upon way.

The group learned to appreciate three major disease subtypes. Since ordinary, hemorrhagic, and modified-spectrum smallpox cases differ with respect to manifestation, death rate, and transmission rate, the distribution of these subtypes in a population could affect disease spread. Accordingly, the group had to pursue related issues, such as the likely prevalence and strength of immunity in people who had been vaccinated long ago. The group also had to agree on probabilities that at any given point a person with smallpox would go to work or school, go to the hospital, or stay home. The question of who continues to circulate is influenced by manifestation and many other factors, and circulation affects not only who is likely to become infected but also who becomes a contact to be traced.

The group developed scenarios for attacks of three different sizes in terms of the number initially infected and size of the community, the site of origin, and characteristics of first cases. Age and household characteristics in the model populations reflect 2000 census data, with communities structured to incorporate homes, neighborhoods, schools, workplaces, and hospitals. Hospital characteristics reflect available U.S. data in terms of service area and population, number of beds, staff with patient contact, and so forth. The group made assumptions about the behavior of healthcare workers, isolation of patients, effectiveness of preexposure vaccine, vaccine efficacy when given at particular points postexposure, and the like.

With respect to overall intervention strategies, the group considered surveillance and containment in quantitative terms, which meant thinking through parameters such as reliability of case ascertainment and efficiency of contact tracing, at various points for various types of contacts. Other possibilities included case isolation; preemptively vaccinating healthcare workers, at various levels of coverage; mass reactive vaccination, at several levels and speeds of coverage; school closings; and other measures, singly or in combination, taking into account that recognizing and confirming the first cases would be slower than with subsequent cases. Decisions such as when to expand a ring vaccination strategy to a wider community would not depend solely on epidemiologic or operational factors, but political or other factors were specifically not considered in the models.

Repeated discussions took place about the details of interventions, smallpox biology, social structures, and other factors. Typically, after a set of assumptions seemed to be in place and modelers had worked with them, related questions would emerge to be discussed, studied, and tried out. A recurring theme was the great difficulty of recognizing, untangling, and reconciling cryptic assumptions.

This sample of factors gives a good idea of the scale and scope of the working group's efforts, enough to support the claim that even if the results of the models were discarded, the process alone would justify the investment. What seem to be fairly close to final results are now emerging for the first two scenarios, and the three models seem to agree in qualitative terms with respect to intervention strategies: essentially, a prompt, thorough surveillance and containment response should be effective. Even that preliminary agreement comes with caveats, however, and exists in general terms, not necessarily in detailed predictions.

The published models should include a great deal of information, enough to allow replication with virtually any desired change in premises, parameter ranges, and scenarios. One aim of publication is to describe the structure, data, assumptions, hypotheses, and logic involved in a clear and comprehensive manner, so that each aspect can be tested, and alternate choices evaluated, by others with experience in the field. This transparency helps guard against the "garbage in, gospel out" phenomenon that plagues some modeling, policymaking, and laboratory and field studies. Peer review is beneficial, but no good substitute exists for active, ongoing involvement of multiple modelers and other experts in the process, even for less critical applications.

Participants in the December 2001 consultation recommended that "analytic modeling become an explicit element in strategic plans for biodefense preparation and response." If that is to happen, the United States needs to develop and sustain appropriate modeling expertise and access. The Working Group on Smallpox Modeling marks a small but important step in that direction. Modeling seems most likely to be of help in strategic planning and preparation (*3*), but the foot-and-mouth disease outbreak in the United Kingdom in 2001 suggests that real-time modeling could also be useful in an infectious disease emergency (*4*).

Fourier is said to have remarked, 200 years ago, that "nature is extremely indifferent towards the difficulties imposed on mathematicians" (*5*). But enormous sums are invested in modeling weather and economies, although the models are often wrong, and too many variables are involved to consistently obtain accurate predictions. Those investments are made because stakes are high: verbal analysis alone cannot provide solutions, and "perfect" models will never appear by magic. The same principle holds true with developing models as policy tools.
